# Unusual Presentation of Leukemic-Phase Mantle Cell Lymphoma: A Case Report

**DOI:** 10.7759/cureus.33239

**Published:** 2023-01-02

**Authors:** Alshaimaa M Alsaati, Meshal M Alasiri, Bayan A Alansari, Wed Y Mulla, Adel F Al-Marzouki, Yara M Daous, Osman Radhwi

**Affiliations:** 1 Medical School, King Abdulaziz University Hospital, Jeddah, SAU; 2 Hematology, King Abdulaziz University Hospital, Jeddah, SAU; 3 Hematopathology, King Abdulaziz University Hospital, Jeddah, SAU

**Keywords:** ccnd1, differential diagnosis, atypical presentation, non-hodgkin’s lymphoma, b-cell lymphoma, mantle cell lymphoma

## Abstract

Mantle cell lymphoma (MCL) is a rare subtype of B-cell lymphoma that can present in a variety of ways, including the leukemic phase, where it can occasionally be mistaken for acute leukemia due to the unusually high or rapidly growing number of leukocytes and the presence of circulating cancer cells that are morphologically similar to leukemic blasts in myeloid or acute lymphoblastic leukemia. We present the case of an 83-year-old Yemeni woman with multiple comorbidities who presented with abdominal pain and constitutional symptoms. She was found to have diffuse lymphadenopathy on clinical and radiological assessments. Her white blood cell count at presentation was 221 × 10^9^/L with marked monocytosis (72.8%). Lymph node biopsy and bone marrow studies, including *CCND1/IGH* molecular studies, confirmed MCL, the pleomorphic subtype. The patient was deemed unfit for standard-of-care chemotherapy and was started on single-agent rituximab with a slow introduction to ibrutinib but succumbed to death after two weeks of ibrutinib 280 mg daily.

This case serves as a reminder to keep an open mind and take into account atypical disease presentations when formulating differential diagnoses to prevent late diagnosis and any unnecessary intervention that can postpone appropriate therapy.

## Introduction

Mantle cell lymphoma (MCL) is an uncommon, incurable, indolent variant of non-Hodgkin lymphoma (NHL) [1]. Despite treatment, MCL acts more aggressively than other mild NHL subtypes [2]. Usually, the age of patients with MCL is around 60-70 years, which is similar to that of patients with diffuse large B-cell lymphoma. It accounts for 5-7% of all types of lymphomas; however, there is a startling sex-distribution imbalance in MCL, with almost 70% of all cases being men [3]. There is less information available regarding MCL risk factors, and significant factors that may contribute to the occurrence of other lymphomas have not been conclusively linked to MCL, with the possible exception of family history [4]. In 80% and 20% of cases, naive B cells and antigen-stimulated B cells are the cells of origin for MCL, respectively [5]. The growth and stability of MCL clones are mediated by complex interactions of cellular microenvironment variables [6]. In the pathogenesis of MCL, the proto-oncogene CCND1 usually expresses as a result of the underlying genetic defect t(11,14)(q13; q32) in all cases of MCL; hence, cyclin D1 is produced more often [5].

In patients with MCL, variable clinical presentations may occur, ranging from asymptomatic lymphocytosis or non-bulky nodal/extranodal disease, with mild symptoms, to progressive generalized lymphadenopathy, cytopenia, splenomegaly, or an extranodal disease affecting different organ systems, such as the kidneys, gastrointestinal tract, and central nervous system (although rarely), with significant symptoms [6,7]. Ascites have been significantly associated with gastrointestinal tract involvement and lesions such as lymphomatous intestinal polyposis, gastric ulcers, and peritoneal lymphomatosis. In addition to ascites, serosal lymphomatosis with pleural and pericardial effusions has been reported in advanced MCL with positive bone marrow or lymph node status. Serosal effusion in malignant lymphomas is uncommon, but it is thought to have a major role in general prognosis and survival [8]. However, the presence of abdominal ascites as a primary presentation of peritoneal lymphomatosis is rare, and only a few cases have been identified in the literature [9].

Due to a high or rapidly growing circulating neoplastic cells and leukocyte count, patients might be mistakenly diagnosed and managed as acute leukemia. In these cases, comprehensive immunophenotypic examination employing a flow cytometric and/or immunohistochemical approach, along with clinical, laboratory, cytogenetic, and radiologic data, may be used to identify NHL [10].

In this report, we discuss a case of primary MCL in an 83-year-old woman who presented with abdominal fullness and weight loss, with marked leukocytosis suggesting either acute leukemia or a leukemic phase of lymphoma.

## Case presentation

We present the case of an 83-year-old Yemeni female patient with a medical history of hypertension, dyslipidemia, diabetes mellitus, atrial fibrillation, and heart failure with a preserved ejection fraction of 60.6%. The patient presented to the emergency department at King Abdulaziz University Hospital on December 25, 2021, complaining of painful mild abdominal distention for four days, loss of appetite for two months, involuntary weight loss, nausea without vomiting, and progressive exertional dyspnea for one year affecting her sleep. She also reported increased painless bilateral lower limb swelling for the past two to three weeks. She noticed multiple painless lumps three years ago but denied any change in size. The patient had no history of peptic ulcer. She was a former shisha smoker but quit smoking 15 years ago and denied any use of alcohol or drugs. The patient had no family history of malignancy or unexpected death.

Upon physical examination, the patient’s vital signs were within normal ranges. She was afebrile with enlarged submental, axillary, and inguinal lymph nodes, which were non-tender, not warm, devoid of skin changes, and hard in consistency. Abdominal examination showed non-tender distention, massive non-tender hepatosplenomegaly, and a non-tender umbilical mass. The chest was clear for auscultation. Pitting bilateral lower limb edema was observed up to the upper shin and was non-tender, with no erythema.

Initial laboratory workup showed marked leukocytosis (221 × 10^9^/L), lymphocytosis (53.56 × 10^9^/L), and monocytosis (161.61 × 10^9^/L). Other laboratory findings were as follows: neutrophils (6 × 10^9^/L), basophils (0.18 × 10^9^/L), eosinophils (0.08 × 10^9^/L), hemoglobin (8.2 g/dL), and platelet count (79 × 10^9^/L). Liver function test results were within normal ranges. Apart from elevated levels of lactate dehydrogenase (1,581 U/L) and uric acid (949 μmol/L) other markers for tumor lysis syndrome, including serum electrolytes and kidney function tests, were within the normal range. The patient tested negative for the human immunodeficiency virus and hepatitis B and C. A blood smear showed abnormal mononuclear cells, atypical lymphocytes, true thrombocytopenia, and a manual blast count of less than 20%.

**Figure 1:**
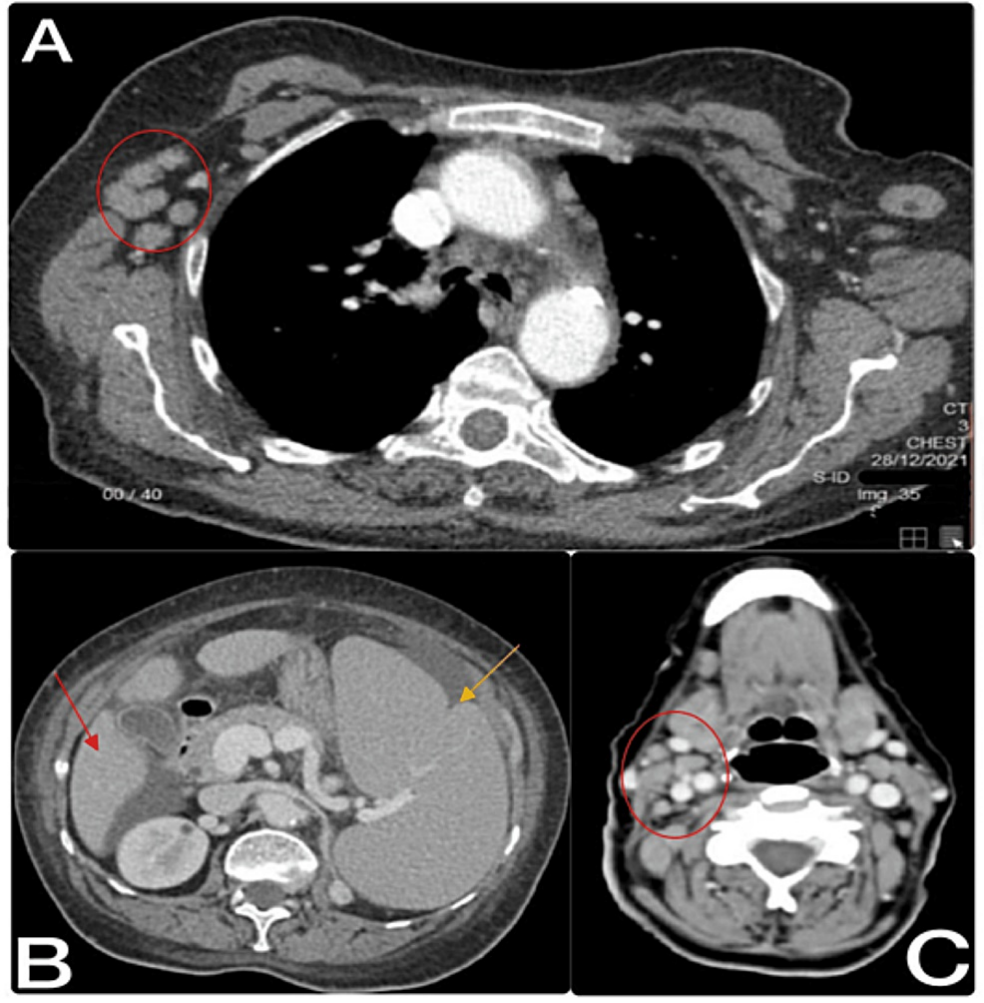
Computed tomography (CT). (A) CT chest showing multiple enlarged axillary lymph nodes (red circle). (B) CT abdomen showing hepatosplenomegaly with minimal ascites (red arrow spleen, yellow arrow liver). (C) CT neck showing multiple prominent upper neck lymph nodes (red circle cervical lymph node).

Body computed tomography (CT) was conducted and showed mainly supra- and infra-diaphragmatic lymphadenopathy, splenomegaly, and a suspicious left adrenal gland lesion (Figures 1A-1C). Brain CT showed no evidence of acute brain insults or space-occupying lesions. Brain magnetic resonance imaging and cerebrospinal fluid analysis were not conducted despite the high central nervous system (CNS) risk because we were considering palliative and non-curative treatment based on patient examination and history, and she did not complain of any symptoms related to the central nervous system (CNS).

Flow cytometry and immunophenotyping of the peripheral blood showed 80% abnormal B cells that were positive for pan B-cell markers (CD19, cytoplasmic CD79a, CD20, and cytoplasmic CD22), CD45 (bright), human leukocyte antigen DR isotype (HLA-DR), CD9, cytoplasmic CD79b, CD10 (dim), and CD200. They were negative for CD3, CD5, CD7, CD34, TdT, CD103, CD38, surface CD22, surface CD79b, FMC7, and CD30. Moreover, examination of 20 metaphases showed a complex karyotype with some missing chromosomes, deletions, and translocations: 42,X,-X,del(1)(q36), t(4;17)(q27;q25),-8, t(12;21)(q24;q22),-14,-18, and del(19)t(9;19)(q11;p11)(20).

**Figure 2:**
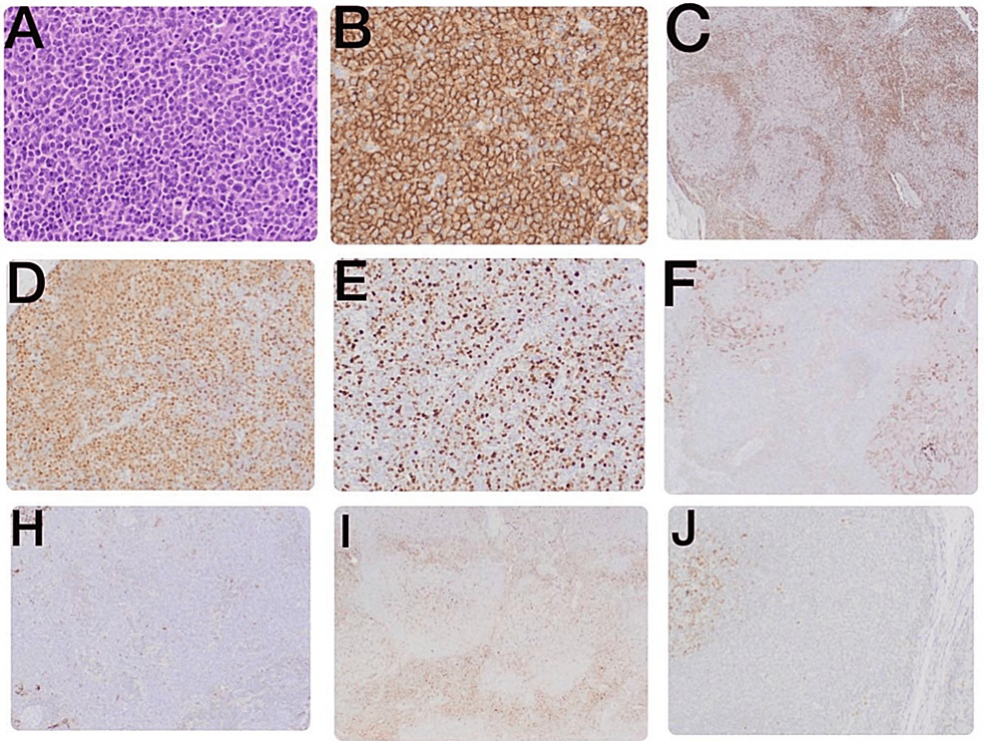
Lymph node (LN) biopsy. (A) LN, hematoxylin and eosin (20×): sheets of pleomorphic, intermediate-to-large lymphocytes with irregular nuclear borders, vesicular chromatin, and prominent nucleoli. (B) CD20, high-power, shows strong and diffuse positivity. (C) CD5 is negative in neoplastic lymphocytes and positive in background bystander T cells. (D) Cyclin D1, high-power, shows strong, diffuse nuclear expression. (E) Ki67, high proliferative rate. (F) CD21, follicular dendritic cell meshwork. (H) CD23 positive in follicular dendritic cell meshwork and negative in neoplastic cells. (I) CD10 negative in neoplastic lymphocytes but positive in few residual reactive germinal centers. (J) BCL-6, positive in reactive germinal center and negative in neoplastic cells.

The patient underwent an inguinal lymph node biopsy (Figure 2A) which showed an architecturally effaced lymph node by vague nodules filled with sheets of pleomorphic intermediate-to-large lymphocytes with irregular nuclear borders, vesicular chromatin, and prominent nucleoli. Mitoses were readily seen. On immunohistochemistry, the neoplastic lymphocytes were positive for CD20 (strong and diffuse), cyclin D1 (nuclear), and BCL-2, while negative for CD5, CD10, and BCL-6. CD21 and CD23 highlighted follicular dendritic cell meshwork but were not co-expressed on lymphocytes. The proliferation index by ki-67 was approximately 25%. Fluorescence in-situ hybridization analysis using a *CCND1/IGH* dual-color dual fusion probe (manufactured by ZytoLight) showed that 27% of cells showed fusions between *CCND1 *and *IGH*. The pathology report was signed out as involvement by a B-cell lymphoproliferative disorder, morphologically and immunophenotypically consistent with CD5-negative MCL, the pleomorphic variant. Overall, 60% of the cells had *TP53 *mutation (Figures 2B-2J).

**Figure 3:**
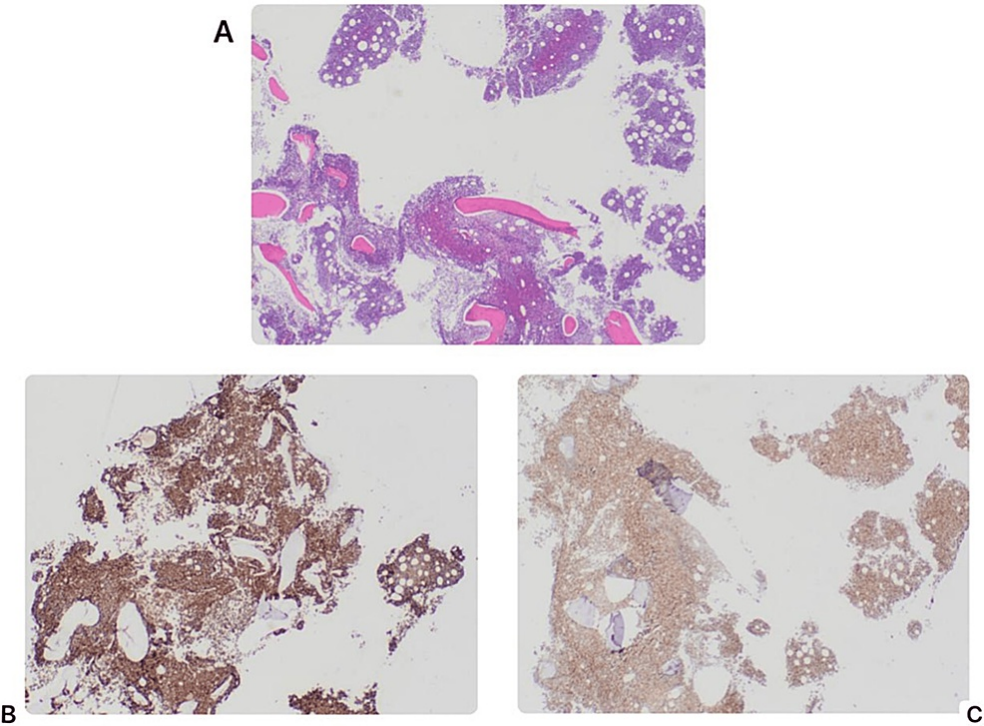
Bone marrow (BM) biopsy. (A) BM hematoxylin and eosin, low power: effacement by a diffuse interstitial lymphoid infiltrate. (B) Mantle BM immunostains, CD20. (C) Mantle BM immunostains, cyclin D1.

The bone marrow biopsy was hypercellular and effaced by a diffuse interstitial lymphoid infiltrate morphologically similar to the one described in the lymph node. Trilineage hematopoiesis was markedly diminished (Figure 3A). A panel of immunohistochemical stains was performed with appropriately reactive controls. The neoplastic lymphocytes were positive for CD20 and cyclin D1 and negative for all the other markers utilized, including CD5 and 138 (Figures 3B, 3C).

The patient was supported with cautious intravenous fluid administration in addition to furosemide, as needed, to avoid exacerbation of heart failure and prevent tumor lysis syndrome. The patient continued to receive bisoprolol 5 mg daily, amlodipine 5 mg daily, perindopril 5 mg daily, atorvastatin 5 mg daily, omeprazole 30 mg daily, and enoxaparin 40 mg daily for venous thromboembolism prophylaxis.

The tumor board discussed the case and agreed on the diagnosis of MCL and the initial treatment plan of single-agent rituximab weekly for four weeks, with escalation to a combination of rituximab and ibrutinib if the patient tolerated the treatment well. She initially received one cycle of rituximab (weekly infusion of 375 mg/m^2^ for four weeks), followed by ibrutinib at a lower dose of 280 mg daily, which was to be increased if well tolerated. The patient showed an excellent early response clinically, as shown by routine laboratory investigations, with the white blood cell (WBC) count returning to normal levels (5.27 × 10^9^/L). However, after the second week of ibrutinib administration, the patient’s condition began to deteriorate clinically and biochemically, with the rapidly rising WBC count (312.91 × 10^9^/L). The patient subsequently developed severe septic shock and, unfortunately, died.

## Discussion

NHLs are diverse malignant tumors of the lymphatic tissue that exhibit unique biochemical, morphological, and clinical features, as well as a broad range of therapeutic responses, prognoses, and patient survival rates. However, most morphological and clinical variations of lymphoma are currently treatable. Most NHLs are generated from tumorous B-lymphocyte analogs, with T-cell and natural killer (NK)-cell malignancies accounting for the remainder. MCL is a B-cell malignancy characterized by small-to-medium-sized monomorphic lymphoid cells with an irregular nucleus [11]. It has been referred to by several names across times, including intermediate lymphocytic lymphoma, centrocytes lymphoma, mantle zone lymphoma, and lymphocytic lymphoma of intermediate differentiation [12,13]. Since the 90s, all of these names have been considered as belonging to the MCL, as it was found that all documented cases had the (11,14) (q13;q32) translocation [5]. MCL can manifest in various ways, from a chronic or mild form to a more severe course that significantly affects overall survival [2]. Hematological malignancies seldom occur with ascites or serous effusions [9], although ascites have been associated with diffuse large B-cell lymphoma, the most common type of NHL. According to a case series, only 8% of 101 patients with malignant ascites and positive cytology findings had lymphomas. In another case series, the rate was lower (2%). Here, we present a case in which MCL was associated with ascites detected through examination and confirmed using CT.

Ascites developing in lymphomas, either primary or otherwise, are considered an adverse factor affecting overall survival; however, our patient responded favorably to diuretics alone, given the small amount of ascites [14-16]. At the time of diagnosis, MCL typically affects the bone marrow, spleen, and gastrointestinal system, in addition to lymph nodes. Frank leukemic involvement at presentation is uncommon; however, when it occurs, it can be difficult to differentiate from B-cell chronic lymphoid leukemia and other chronic lymphoproliferative diseases such as follicular lymphoma in the leukemic phase. This differentiation is crucial because MCL frequently pursues an aggressive clinical course with a total life expectancy of only three to five years [17]; the leukemic phase in classic MCL has been described in one case report [18,19]. Our patient presented with a high mitotic rate, peripheral blood lymphocytosis, and an aggressive clinical course; thus, distinguishing the disease from acute leukemia was challenging. As previously mentioned, flow cytometric immunophenotyping has become crucial to the lymphoma diagnostic workup. In addition to expressing pan-B-cell antigens, MCLs typically have a CD5+/CD10- phenotype, and variations from the typical immunophenotype in MCL have been observed in a few cases. As we found in the literature, unusual presentations with a CD5-/CD10+ immunophenotype, as in our case, are rare, which can cause considerable diagnostic struggle and may lead to a wrong diagnosis of classical MCL. This further underlines the significance of investigating cyclin D1 expression and/or the presence of t(11;14) in a proper morphologic setting to determine an appropriate subclassification of B-cell lymphoma [20].

Currently, there is no established first-line therapy for MCL. Several regimens prolong response times; however, none are curative. The current approach to treating patients is based on the patient’s age and level of fitness, with younger or more physically fit patients receiving intensive combination therapies, including rituximab and cytarabine, with or without consolidation autologous stem cell transplantation, and older or unhealthy patients receiving combination chemotherapy and immunotherapy, with or without rituximab maintenance. Emerging approaches combine more recent therapies, such as ibrutinib, in frontline therapy and use prognostic indicators, such as minimal residual disease, to guide medication decisions [21].

Owing to the various comorbidities that our patient reported, we determined that combination therapy with rituximab and ibrutinib was the most effective.

## Conclusions

We present a case of mantle cell lymphoma with leukemic-phase features, namely, high WBC count and lymphocytosis. These characteristics likely had a significant impact on the disease’s clinical progression and affected the overall survival rate. Proper diagnosis and management of such cases along with follow-up will improve patient survival and reduce complications in the future.
